# Implementation and Validation of a Two-Stage Energy Extraction Circuit for a Self Sustained Asset-Tracking System †

**DOI:** 10.3390/s19061330

**Published:** 2019-03-16

**Authors:** Philipp Dorsch, Toni Bartsch, Florian Hubert, Heinrich Milosiu, Stefan J. Rupitsch

**Affiliations:** 1 Chair of Sensor Technology, Friedrich-Alexander-University, Paul-Gordan Str. 3/5, 91052 Erlangen, Germany; florian.hubert@fau.de (F.H.); stefan.rupitsch@fau.de (S.J.R.); 2 Department of Integrated Circuits and Systems, Fraunhofer Institute for Integrated Circuits, Am Wolfsmantel 33, 91058 Erlangen, Germany; toni.bartsch@fau.de (T.B.); heinrich.milosiu@iis.fraunhofer.de (H.M.)

**Keywords:** energy harvesting, piezoelectricity, energy extraction networks

## Abstract

We present a two-stage energy extraction circuit for a piezoelectric energy harvester, powering an asset-tracking system. Exploiting accelerations generated by many logistic transport devices, e.g., pushcarts, forklifts, assembly belts or cars, we are able to harvest sufficient electrical energy to transmit radio signals, which will allow to track an object when it is moving. Accelerations in logistic applications are non-sinusoidal and lead to high open-circuit voltages, which demand a special adaption of the energy extraction network. We evaluate the performance of several state-of-the-art energy extraction networks and compare those to the performance of our two-stage approach under various excitation conditions. By using the proposed energy extraction circuit, the transmission rate could be increased from four to six transmissions per second for sinusoidal excitations with an open-circuit-voltage of 60V. In the practical use-case, the two-stage energy extraction network performs more than two times better compared to the one-stage and synchronized switching harvesting with inductor approach.

## 1. Introduction

Nowadays, wireless sensor systems for consumer, industrial or medical applications are mostly powered by batteries. Those have to be replaced or recharged after they are discharged and they have to be disposed after their lifetime. Depending on the assembly situation, this can not only be inconvenient in terms of cost efficiency but also harmfull for the environment [1]. Consequently, there is a great demand for self-sustained systems, which do not require batteries. The key-technology for the development of such systems is energy harvesting [2,3].

Generally speaking, the energy harvesting technology has to be chosen according to the desired use-case, providing the electrical power demand of the application under the conditions of the considered excitation [4]. For many outdoor applications, photovoltaic cells are the preferable energy harvesting technique, as discussed in Section 2. They feature high power densities as well as easy and cost efficient implementation [5]. However, if one can not rely on the presence of power from solar radiation, other harvesting techniques might be preferred. In case of excitation by mechanical vibrations, for instance, piezoelectric materials deliver an excellent conversion rate from mechanical into electrical power [6].

However, this is not the only reason why piezoelectric materials received great attention by researchers in the last decades. The output signal of PEHs can not only serve as a source of power for operating low-power sensors and radio frequency transmission circuitry, but can also be used as a sensor. This “smartness” is exploited for various applications, e.g., health monitoring of mechanical structures, like bridges [7], railway tracks [8], and aeronautical applications [9]. Piezoelectric materials are not only suitable for integration [10] in such mechanical structures but also for designing miniaturized energy harvesting devices with low resonance frequency (30Hz) [11]. Possible applications also include the harvesting of wind energy [12].

Generally speaking, piezoelectric materials feature high open-circuit voltages and a high internal impedance, which makes the design of a proper energy extraction network a tedious task. It has to be customized according to the excitation generated by the desired use case as well as adapted to the electro-mechanical coupled behavior of the piezoelectric energy harvester. Furthermore, it has to supply the harvested energy at a defined voltage according to the demands of the electrical consumer.

The application to realize is an energy autarchic asset tracking system [13,14,15]. Generally speaking, this idea is not new since there are several approaches, which investigate the feasability of realizing an energy autarchic tracking modul, e.g., for tracking cars [16]. However, the amount of electrical energy required for operating a gps/gsm modul in order to acquire and send the position equals ≈120J, which needs 3.2 h of continuous driving to be harvested. For indoor asset-tracking applications, Radio-Frequency-Identication (RFID)-tags are conventionally used. Passive RFID-tags can be read out only in a short distance of up to 30cm since the electrical energy to power the tag is transfered inductively, provided by the RFID-reader. For some tasks (e.g., detecting the passing of a gate), this range is not sufficient. Active RFID-tags with backscattering technology can be read out in a distance of 2–10 m, but such systems require electrical power of up to hundreds of mW in sending-mode and a few μW in sleep-mode [17]. A commercially available lithium button cell (e.g., Li-Mn, CR2025, 3 V, 165 mAh) with an electrical energy amount of 1500J provides enough energy for a few million radio transmissions. Reading the RFID-tag once every minute results in a battery-lifetime of 2–4 years. However, the proposed asset-tracking-application demands higher update rates of one transmission per second, which would result in an unacceptable battery-lifetime of only two weeks. The feasibility of extending battery lifetime of such tags by means of the piezoelectric energy harvesting was already investigated with a battery-supported one-stage approach in Ref. [5].

In contrast to the mentioned approach, our proposed self-sustained asset tracking system harvests the power solely from mechanical vibrations, which arise from the movement of the asset by means of the piezoelectric effect. This is meaningful because the mechanical input power does not only serve as a source of energy but also as a trigger for the radio transmissions. Receiving those signals with stationary receiver beacons, it is possible to calculate the position of the object-to-be-tracked [18]. In other words, the realized system is not only completely self-sustained, but also features a movement-dependent position update rate. Together with the benefit of not having to change and recycle batteries, this is another great advantage compared to battery powered tracking systems, since the update rate of such systems has to be chosen as a tradeoff with battery life. The concept of the system is explained more detailed in Section 2.

Section 3 describes the design and manufacturing process of the developed piezoelectric energy harvester. In Section 4, the considered excitation and the behavior of the piezoelectric energy harvester (PEH) are characterized. As a matter of fact, PEHs are electromechanically coupled systems [2,19]. To extract the maximum electrical power of such systems, both the mechanical excitation and the electrical load have to meet the conditions of the maximum power point (MPP) of the PEH. On the one hand, it is essential to characterize the surrounding acceleration power spectrum of the considered use-case to be able to adapt the mechanical side of the PEH accordingly. On the other hand, the optimal electrical load has to be connected to the piezoelectric energy harvester to extract the maximum electrical power from it [20].

In Refs. [21,22,23,24], different energy extraction schemes are discussed that deal with this issue. Section 5 compares the performance of passive energy extraction networks like full bridge rectifiers and active energy extraction techniques like Synchronized Switching Harvesting with Inductor (SSHI). Furthermore, the proposed two-stage energy extraction circuit is introduced and its performance is compared to the one-stage approach and the SSHI-approach. This is evaluated for sinusoidal and noise excitation conditions on a vibration test system (VTS) and in situ for the desired usecase.

The two-stage approach comprises two DC/DC-converters. The first-stage consists of an input voltage controlled flyback-converter and provides proper electrical load conditions to the PEH. The second stage is a low power buck-converter, which is only active while sending a radio transmission. It provides a regulated output voltage for the application.

Another two-stage design was proposed by Ref. [25] and consists of a custom application specific integrated circuit design with ultra low power linear drop out regulator as second stage. In contrast to this implementation, we use only comercially available components and a buck converter as second stage, which features an efficiency of 85–90% for an input voltage of 10–4 V [26] and gets only activated in sending mode. Due to the discrete implementation, our concept can be easily adapted to different scenarios. We were able to power the microcontroller transmitter combination and achieved a median transmission rate, which is about two times higher, compared to the one-stage approach or the SSHI-approach.

## 2. Concept of the Self-Sustained Asset-Tracking System

For a comparison of the performance of several energy conversion mechanisms, batteries and accumulators, with respect to their lifetime and volumetric power densities, a brief overview is given in Refs. [14,27,28,29]. Since the goal of this contribution is to build a self-sustained asset tracking system with a long lifetime, which does not rely on the presence of radiation power through direct sunlight, the piezoelectric conversion principle was chosen.

**Figure 1:**
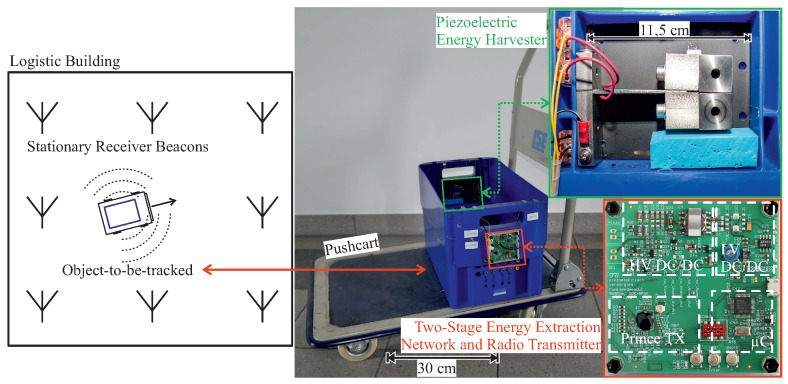
(**Left**): Function principle of the autarchic asset tracking system. (**Right**): Piezoelectric energy harvester and the two-stage energy extraction network are included in the object-to-be-tracked, which is placed on a pushcart.

The realized asset tracking system is depicted in Figure 1, where the PEH is mounted in a storage container, which is the object-to-be-tracked. So the exploited power source are mechanical vibrations, that arise from the movement of the logistic container. The PEH is connected to the energy extraction network, which accumulates the harvested electrical energy until there is enough to send one radio transmission. For the used microcontroller transmitter combination, the required amount of electrical energy is 380μJ, provided at 3.3V. After this, the microcontroller (STM32LO) and the Prince-Transmitter are activated and generate a 868MHz radio signal, modulated with On Off Keying (OOK) or Binary Phase Shift Keying (BPSK), which contains a unique identification number of the object-to-be-tracked. Receiving this signal with stationary signal beacons, it is possible to calculate the position of the sending object by a joint angle and delay estimation [18].

The resulting system is not only completely self-sustained, but also features a movement dependend position update rate. Needless to say, the concept can be scaled to the required amount of energy.

## 3. Design and Manufacturing Process of the Cantilever-Based Piezoelectric Energy Harvester

**Figure 2:**
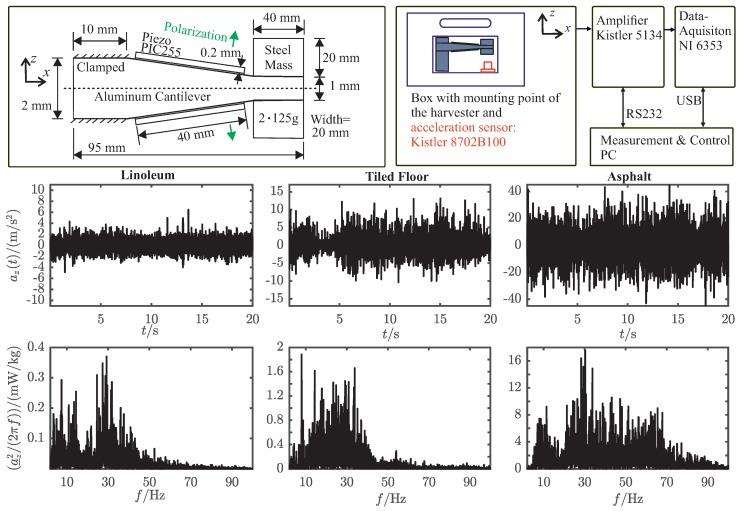
(**Top left**): Geometric dimensions of the piezoelectric energy harvester. (**Top right**): Measurement setup of acceleration measurement. (**Mid**): Measured transient acceleration $a_{z}\left(t\right)$ of a pushcart driven on different undergrounds. (**Bottom**): Power spectrum ${\underline{a}}_{z}^{2}\left(f\right)/\left(2\pi f\right)$ of the measured transient accelerations.

For the cantilever material, we choose aluminum because it is easy to machine and comprises a low dissipation factor (tan(δ) = 0.0016) [14], compared to glass- or carbon-fiber composites. The aluminum cantilever features a wedge shape (Figure 2), which contributes to a more uniform distribution of mechanical stresses in the piezoelectric material, and thus leads to a better performance. It is designed according to analytical methods, finite element simulations and measurements, which are discussed in previous publications [13,14,30]. Moreover, a special modal-reduction technique was used [19] in order to perform efficient transient simulations of the PEH and the electrical energy extraction network.

The wedge shape of the aluminum cantilever was manufactured using a conventional milling machine and a CNC-milled holding plate, which ensures the right slope for the two milling operations of each side and enables secure clamping in a vice. Mounting holes make it possible to attach the device in a defined way to the clamping in the logistic box and the vibration test system. This is essential in order to get reproduceable results for the in situ and characterization measurements of the harvester.

For the piezoelectric material, several choices are possible. Flexible polyvinylfluorid (PVDF)-based materials received great attention by researchers [10,11,12]. Due to their high piezoelectric constants ($d_{31}=25\;pC\;N{}^{-1}$ [31]) and a low relative dielectric permittivity ($\varepsilon_{33}/\varepsilon_{0}\approx$ 9–28 [32]), they provide higher electrical open-circuit voltages but also a much higher inner impedance, compared to lead zirconate titanate (PZT) materials. Thus, the choice of the piezoelectric material is not an easy task, which is why several figures of merit were discussed and introduced in Ref. [33].

According to those figures of merit, two plates lead zirconate titanate with a size of 40mm×20mm×0.2mm, polarized in thicknes direction and electrically connected in series are used. We choose the piezoceramic material PIC255 (PI-Ceramic GmbH, Lederhose, Germany) because of its high piezoelectric strainconstant ($d_{31}=-174\;\times \;10^{-12}\;m\;V{}^{-1}$) and excellent coupling coefficient ($k_{31}=0.35$) [6]. Moreover, a reliable set of material parameters has been evaluated for PIC255 [34,35,36,37], which was essential for the finite element simulation based design approach of this harvester [13].

After sanding and cleaning the faces of the aluminum cantilever, the two piezoelectric plates were bonded to it using two types of two-component epoxy-adhesive. Masking the glue area on the aluminum with adhesive tape, it was possible to squeegee the epoxy-glue in a defined height. A small area of conductive epoxy (2mm×2mm) was located in the middle of the piezoelectric element to ensure conductivity between the inner electrodes of the piezoelectric elements and the aluminum cantilever. For the remaining part of the adhesive area, conventional two-component epoxy glue (UHU plus Endfest 300, 100 weight parts of binder and 50 weight parts of hardener) was applied in the same mannor. The viscosity of the adhesive mixture was adjusted by heating it up to be able to squeedgee it properly. After fixing the position of the ceramic with adhesive-tape, we applied pressure by clamping the PEH into a vice inbetween two silicon plates. The whole setup cured for 60min at $80{\;}^{{}^{\circ}}C$ in a clima chamber and remained in it, allowing it to cool down to avoid thermal stresses. The resulting piezoelectric energy harvester is depicted in Figure 2.

## 4. Characterization of the Excitation and the Piezoelectric Energy Harvester

**Figure 3:**
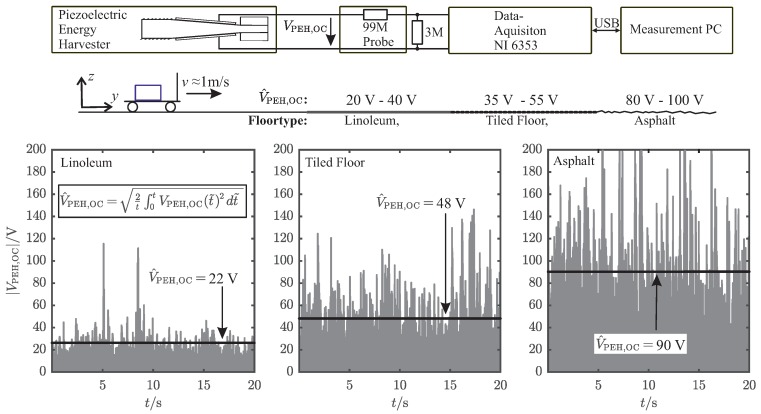
(**Top**): Measurement setup of the open-circuit voltage measurements. (**Bottom**): Transient absolut value of the open-circuit voltages $|{\hat{V}}_{\mathrm{PEH},\mathrm{OC}}|$ from the PEH for driving with the pushcart on different floortypes.

To extract the maximum electrical power of piezoelectric energy harvesters (PEH), both the mechanical excitation and the electrical load have to be at the maximum power point (MPP) of the PEH. Therefore, it is essential to characterize the surrounding acceleration power spectrum of the considered use-case to be able to adapt the mechanical side, the resonance frequency, of the PEH accordingly. The measurement setup for acquiring this data and the results are depicted in Figure 2. The measurements were carried out without the PEH mounted to the logistic box, since it would influence the measured spectrum by absorbing mechanical power around its resonance frequency. Hence, it was not possible to acquire acceleration and open-circuit voltage measurements (Figure 3) at the same time. We measured the acceleration $a_{z}\left(t\right)$ of the pushcart driving on several undergrounds and calculated the power spectrum with

(1) $$P_{\mathrm{acc}}\left(f\right)=\underline{a}{\left(f\right)}^{2}/\left(2\pi f\right);\;\left[P_{\mathrm{acc}}\left(f\right)\right]=W\;kg{}^{-1}.$$

$P_{\mathrm{acc}}\left(f\right)$ denotes the spectral mechanical input power that a free mass would receive in a sourrounding acceleration field, which is used as a measure of the mechanical input power of the system. Even though there is no distinct peak frequency detectable, it can be stated that the most surrounding power is at the frequency range around 30 Hz for several usecases, as shown in Figure 2.

**Figure 4:**
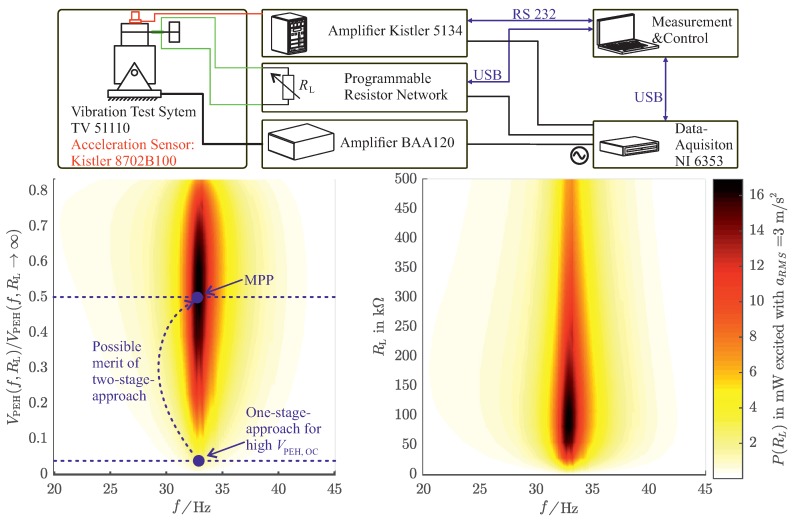
(**Top**): Measurement setup for the power output of the PEH for sinusoidal excitation with a vibration test system. (**Right**): Power output with respect to a directly connected load resistance $R_{L}$. (**Left**): Power output with respect to the frequency dependent open-circuit voltage $V_{\mathrm{PEH}}\left(f,R_{L}\to \infty \right)$. The arrow indicates the possible merit of the two-stage approach by providing better electrical load conditions to the PEH.

In the next step, the mechanical and electrical behavior of the PEH were evaluated. Figure 4 shows the measurement setup and the measurement results for the characterization of the PEH. The setup is excited with respect to sinusoidal accelleration with $a_{\mathrm{rms}}=3\;m/s^{2}$. Although this accelleration value might seem to be small compared to those of the noise excitation, it was chosen in order to do not destroy the PEH when exciting it sinusoidally at its resonance frequency $f_{r}=\;33\;\mathrm{Hz}$. It can be seen that the electrical output power $P(f,R_{L})$ depends on the excitation frequency *f* and the electrical load resistance $R_{L}$, which is directly connected to the PEH. The resonance frequency of the PEH was designed to be in the aforementioned frequency range. Therefore, the PEH can gather the ambient mechanical power efficiently for the desired usecase. For an electrical load of $R_{L}\approx 100\;k\Omega$, the electrical output power is at its maximum. This power can also be interpreted with respect to the ratio of the PEH output voltage $V_{\mathrm{PEH}}\left(f,R_{L}\right)$ under electrical load and the PEH open-circuit voltage $V_{\mathrm{PEH}}\left(f,R_{L}\to \infty \right)$. The optimal electrical load corresponds to the ratio $V_{\mathrm{PEH}}\left(f,R_{L}\right)/V_{\mathrm{PEH}}\left(f,R_{L}\to \infty \right)\approx 0.5$.

As a matter of fact, PEHs produce much higher open-circuit voltages compared to the supply voltages needed for low-power integrated circuits. Considering the desired usecase, for instance, the PEH delivers an alternating voltage of ${\hat{V}}_{\mathrm{PEH}},\mathrm{OC}\approx 90\;V$ (Figure 3). However, the microcontroller-transmitter unit requires a distinct supply voltage of 3.3V as well as a certain amount of energy to operate correctly. If we consider a simple rectifier energy extraction network loading a storage capacitor, which works around the desired supply voltage of 3.3V, this will result in a ratio of $V_{\mathrm{PEH}}\left(f,R_{L}\right)/V_{\mathrm{PEH}}\left(f,R_{L}\to \infty \right)=0.04$. Such a low value implies a total mismatch of the optimum electrical load. The measurement results in Figure 4 reveal that only about 1/16 of the maximum available power can be extracted for this operating point. The two-stage energy extraction network, which is introduced in Section 5, provides a much higher ratio of $V_{\mathrm{PEH}}\left(f,R_{L}\right)/V_{\mathrm{PEH}}\left(f,R_{L}\to \infty \right)$ and therefore a better power extraction.

## 5. The Two-Stage Energy Extraction Network

Generally speaking, energy extraction networks can be divided in passive and active techniques: passive circuits, like full bridge rectifiers or voltage doublers, feature easy and cost-efficient implementation and do not need electrical power to supply themselves. Moreover, they do not rely on information about the open-circuit-voltage or any other mechanical properties (e.g., tip-displacement) of the PEH and do not fail for non-sinusoidal excitations. To explain the restrictions of passive energy extraction techniques, we consider a PEH connected to a rectifier charging a capacitor $C_{1}$. In case of high open-circuit voltages, the forward voltages of the diodes can be neglected and with $C_{1}\gg C_{\mathrm{PEH}}$, the capacitor $C_{1}$ can be interpreted as voltage source with the voltage $V_{C1}$. The extractable energy $W_{E}$ and its maximum results in

(2) $$W_{E}\left(V_{C}1\right)=\left({\hat{V}}_{\mathrm{PEH},\mathrm{OC}}-V_{C1}\right)V_{C1}C_{\mathrm{PEH}}\to \max\left(W_{E}\right)=W_{E}\left(V_{C1}=0.5{\hat{V}}_{\mathrm{PEH},\mathrm{OC}}\right)=\frac{1}{4}{\hat{V}}_{\mathrm{PEH},\mathrm{OC}}^{2}C_{\mathrm{PEH}}.$$

Since the energy extraction is done by charge exchange of two capacitors ($C_{\mathrm{PEH}}$ and $C_{1}$), the ratio of extractable energy is restricted to 50% of the maximum electrical energy in the PEH capacitor [14,38].

**Figure 5:**
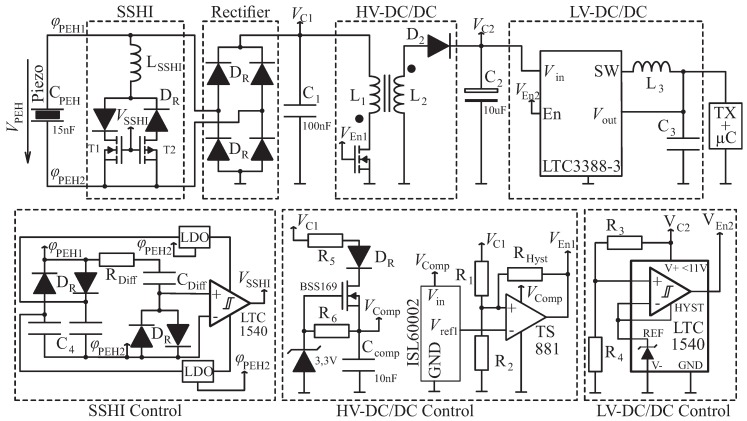
Schematic of the two-stage energy extraction network. (**First row**): Power section with parallel SSHI unit, High Voltage DC/DC (HV-DC/DC) converter in flyback-topology, and Low Voltage DC/DC (LV-DC/DC) in buck-topology. (**Second row**): Control-circuit section with SSHI Control, HV-DC/DC Control, and LV-DC/DC Control. Parts of the circuit can be disabled and enabled, which results in different energy extraction networks, refer to Table 1 for an explanation of the different network modes.

Active topologies, like SSHI [22,23] and Synchronized Electric Charge Extraction (SECE) [24], pursue to maximize the extractable energy $W_{E}$, by connecting an inductivity at the maximum tip deflection of the PEH. In practical usecases, this point is detected by the maximum of the PEHs open-circuit voltage. This is a challenging task because the electric control circuit should be designed with components that require low power or no power at all to run. Examples are shown in [3,39,40] and depicted in Figure 5, refered to as SSHI control. Since the control circuit consists of a differentiator ($R_{\mathrm{Diff}},C_{\mathrm{Diff}}$), exciting the PEH with non-sinusoidal accelerations can result in unwanted switching behavior of the control circuit, which causes a bad performance of active energy extraction networks.

Moreover, even in the case of sinusoidal excitation, active energy extraction techniques are not performing better than passive energy extraction techniques for PEHs with high coupling coefficients that are excited with reference to ambient acceleration. This is congruent with the results published in this contribution and also stated in [21,22]. The reasons for this astonishing behavior originate from the inverse piezoelectric effect (backward-coupling), which is not covered by modelling the PEH as a current source connected to a capacitor. For piezoelectric materials with high coupling coefficients, switching events of active energy extraction techniques excite higher mechanical modes, which lead to additional dissipation [41,42,43].

Figure 5 shows all parts of the considered energy extraction networks. Those parts can be enabled and disabled, which results in different energy extraction topologies. Table 1 gives an explanation of the different network modes. In the following, the two-stage energy extraction approach is introduced. Therefore, SSHI and its corresponding control circuit are disabled.

The first stage, which is refered to as “High Voltage-DC/DC Converter” (HV-DC/DC), of the proposed energy extraction circuit is realized as a full bridge rectifier connected to a capacitor $C_{1}$. In order to regulate $V_{C1}$, a low side flyback converter topology was chosen. Because all switching elements can be controlled with reference to one common ground potential and no negative supply voltages are required, there is no need for additional voltage level shifters. This results in an easier implementation and less electrical power consumption of the control circuitry. The HV-DC/DC regulates its input voltage $V_{C1}$ so that it remains at predefined high voltages (10–50V) where the energy extraction efficiency is higher. The regulation value of $V_{C1}$ can be adjusted with the voltage devider $R_{2}/\left(R_{1}+R_{2}\right)$. In other words, the purpose of the HV-DC/DC is to provide proper electrical load conditions to the PEH, which also means keeping the ratio of $V_{\mathrm{PEH}}\left(f,R_{L}\right)/V_{\mathrm{PEH}}\left(f,R_{L}\to \infty \right)$ closer to 0.5, according to Figure 4.

Since the HV-DC/DC regulates its input voltage $V_{C1}$, its output Voltage $V_{C2}$ is unregulated. However, most electrical applications, like the considered transmitter microcontroller combination, demand a certain amount of electrical energy provided at a defined electrical voltage (e.g., 3.3V) to perform their task correctly. This is the purpose of the second stage, which is refered to as “Low Voltage DC/DC” (LV-DC/DC), according to Figure 5. The capacitor $C_{2}$ stores the electrical energy for the application. It is charged by the HV-DC/DC until it reaches the switching Voltage $V_{\mathrm{sw},\mathrm{on}}=9.5\;V$ of the LTC1540 comparator. The comparator enables the LTC3388-3 buck converter, which outputs a regulated voltage $V_{C3}=3.3\;V$ and powers the desired application. When $V_{C2}$ reaches the off-switching value $V_{\mathrm{sw},\mathrm{off}}=3.8\;V$, the buck converter will be disabled and $C_{2}$ will be charged again. The required amount of electrical energy to power the desired application can be scaled by $C_{2}$, according to

(3) $$W_{elec,App}=\frac{1}{2}C_{2}\left(V_{\mathrm{sw},\mathrm{on}}^{2}-V_{\mathrm{sw},\mathrm{off}}^{2}\right)=\frac{1}{2}\;\cdot \;10\;\mu F\;\cdot \;\left[{\left(9.5\;V\right)}^{2}-{\left(3.8\;V\right)}^{2}\right]=380\;\mu J,$$

where the switching voltages $V_{\mathrm{sw},\mathrm{on}}=9.5\;V$ and $V_{\mathrm{sw},\mathrm{off}}=3.8\;V$ are chosen with respect to the maximum supply voltage of the LV-DC/DC comparator and the minimum input voltage of the buck-converter. The voltage transient $V_{C2}$ for one transmission is depicted in Figure 7.

Concluding, the proposed two-stage energy extraction circuit is able to provide good electrical load conditions for the PEH with the HV-DC/DC and also achieves to supply the application at a defined electrical voltage and the required amount of electrical energy with the LV-DC/DC.

### 5.1. Loss-Consideration of the HV-DC/DC

Since the HV-DC/DC is the only part of the circuit, which is permanently active, it has the most influence on the efficiency of the proposed energy extraction network. Other parts of the circuitry, e.g., the LV-DC/DC and the microcontroller transmitter circuitry, are only active in transmission mode. The efficiency for the steady state of the LV-DC/DC (LTC3388-3), working with an input voltage range of $V_{C2}=\left(9\;-\;4\right)\;V$ and a load current of 50mA, accounts for (88–93)% [26].

**Figure 6:**
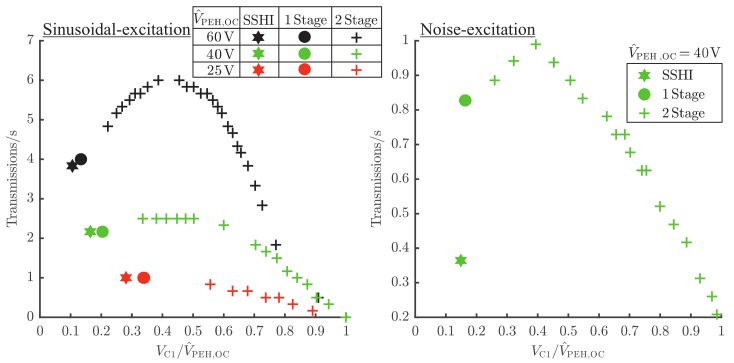
Comparison of the transmission rates for SSHI-, one-stage and two-stage energy extraction networks. (**Left**): sinusoidal-excitation (**Right**): noise-excitation; measured for different acceleration values, which correspond to different open-circuit voltages ${\hat{V}}_{\mathrm{PEH},\mathrm{OC}}$. The results are obtained from the measurement setup in Figure 4, where the resistor network is supplemented with the proposed energy extraction network in Figure 5. To adjust the regulated value of $V_{C1}$, the voltage devider $R_{2}/\left(R_{1}+R_{2}\right)$ is varied in order to evaluate the performance for different ${\hat{V}}_{\mathrm{PEH},\mathrm{OC}}/V_{C1}$.

**Figure 7:**
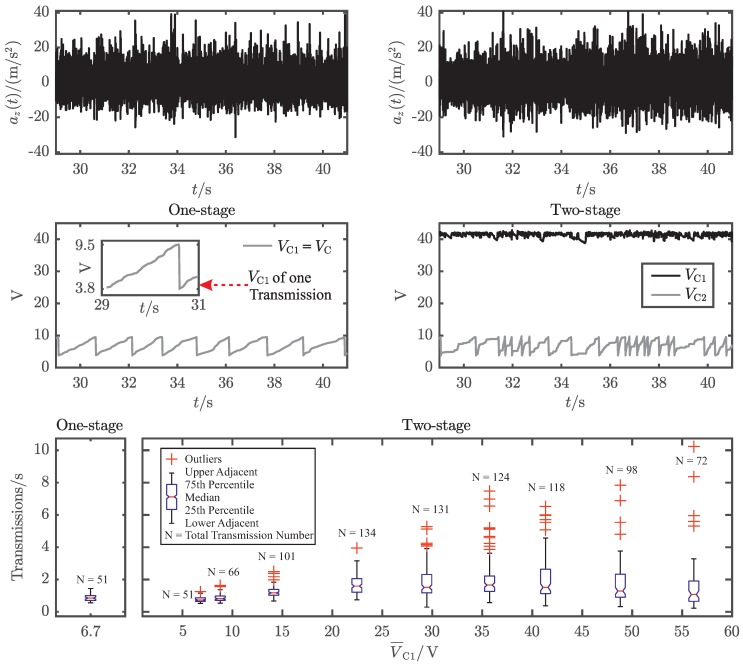
Comparison of the one-stage and two-stage energy extraction network (Figure 5), connected to the PEH in the logistic container on a pushcart (Figure 1) driven on asphalt street. Table 3 shows the corresponding values of this measurement results.

Of course, energy extraction networks have to be as simple as possible, and their benefit has to be greater than their energy consumption. For high open-circuit voltages, the adaption of ${\bar{V}}_{C1}$ to higher values leads to a much higher amount of extractable energy or power than the losses caused by the additional circuitry. This is shown by the possible merit of the two-stage approach, displayed in Figure 4 and in the results measured for exciting the harvester with the vibration test system (Figure 6) as well as for the in situ measurements (Figure 7).

We evaluated several configurations for the design of the HV-DC/DC and considered three different combinations of the inductive component and comparators. With a view to minimizing the volume of the inductive component, a more dynamic comparator (TS881) had to be used. In doing so, the switching time $t_{\mathrm{on}}$ and the amount of input energy $E_{\mathrm{in}}$ per switching period is reduced and the size of the inductive component can be reduced accordingly. The results for the efficiency calculation per period are given in Table 2. We were able to minimize the volume of the required inductive component, while maintaining the efficiency at the same time. Thus, the ER11 core without airgap and the TS881 comparator are used for the realization of the HV-DC/DC.

### 5.2. Performance of the Proposed Network Excited with a Vibration Test System

To evaluate the performance of the energy extraction networks under defined excitation conditions, it is excited with the vibration test system (VTS), depicted in Figure 4. As a measure of the performance, the transmission rate is chosen. It can also be interpreted as mean usable power for the application. The performance was evaluated for several energy extraction network types, Table 1 explains the configurations.

For the one-stage case, the HV-DC/DC, the SSHI and the corresponding control circuits are disabled so that only the LV-DC/DC is active. In this configuration,

(4) $$V_{C1}=V_{C2}=V_{C}\;\;\;\mathrm{so}\;\;\mathrm{that}\;\;{\bar{V}}_{C}=\frac{V_{\mathrm{sw},\mathrm{on}}+V_{\mathrm{sw},\mathrm{off}}}{2}.$$

The vibration test system excites the PEH with sinusoidal acceleration signals of different magnitude $\hat{a}$ close to the resonance frequency $f_{r}$ of the PEH and with noise signals. This leads to different open-circuit voltages ${\hat{V}}_{\mathrm{PEH},\mathrm{OC}}$, which were calculated according to $\sqrt{2}\;\cdot \;V_{\mathrm{PEH},\mathrm{OC},\mathrm{RMS}}$ for both noise and sinusoidal exciations. For the two-stage measurements, $V_{C1}$ is varied by adjusting the voltage devider $R_{1}/\left(R_{1}+R_{2}\right)$. Figure 6 shows the results of the measurements:

The SSHI and the one-stage approach perform equivalent for sinusoidal excitations. For noise-excitation, the one-stage approach delivers a transmission rate, which is more than double of the SSHI-approach. The results for the sinusoidal excitations are congruent with those in literature [21,22], when refering to PEHs with high coupling coefficient that get excited with reference to ambient acceleration. For excitation with noise signals, the SSHI control circuit produces unwanted switching events, which leads to significant performance loss.

Comparing the performance of the one-stage and the two-stage approach, the results show the expected behavior, according to Figure 4. For low excitation or low open-circuit voltages ${\hat{V}}_{\mathrm{PEH},\mathrm{OC}}=25\;V$, respectively, the two-stage approach does not perform better than the simple one-stage approach. This is so because the ratio of ${\bar{V}}_{C1}/{\hat{V}}_{\mathrm{PEH},\mathrm{OC}}$ is close to the optimum condition already, and therefore there is no need for adapting $V_{C1}$ to higher values. However, for higher open-circuit voltages (e.g., 60V), there is a significant improvement of the transmission rate of about 50% for the sinusoidal excitation and 20% for the noise-excitation. In the next section, the performance of the proposed energy-harvesting powered tracking system is evaluated for the desired usecase.

### 5.3. In Situ Evaluation for the Desired Usecase

The system performance is evaluated using the test setup, which is depicted in Figure 1 with the PEH connected to the energy extraction network, shown in Figure 5. The different configurations of energy extraction networks and PEH can not be measured simultaniously. This is so because two identical PEHs mounted in the same box would influence each other as well as the exciting acceleration spectrum and therefore lead to erronous results. To provide realistic as well as comparable test conditions, the cart is pulled by a person at normal walking speed ($v\approx 1\;m\;s{}^{-1}$) while the transient acceleration $a_{z}\left(t\right)$ is recorded. Furthermore, the electrical voltages $V_{C1}$ and $V_{C2}$ are measured. For the two-stage approach, $V_{C1}$ is varied with the voltage devider $R_{1}/\left(R_{1}+R_{2}\right)$. The measurement results are presented in Figure 7. The top and middle part of this figure show two comparable transient accelerations $a_{z}\left(t\right)$ and the corresponding transient voltages $V_{C1}$ and $V_{C2}$, for the one-stage and the two-stage approach. Due to the statistical nature of the exciting accelerations, the measurement results were evaluated with respect to the reciprocal values of the time between two consecutive radio transmissions and visualized in a box-plot (Figure 7).

The achieved transmission rate for the one-stage approach is between $1.44\;s{}^{-1}$ and $0.55\;s{}^{-1}$ with a median value of $0.85\;s{}^{-1}$ (Table 3). This corresponds to a mean usable electrical power of 0.85Transmission/s·380μJ=323μW. The two-stage approach produces much higher outliers for high voltages of ${\bar{V}}_{C1}$, e.g., 10.2Transmission/s for a voltage of ${\bar{V}}_{C1}=56.2\;V$. This is caused by the statistical behavior of the acceleration signal or the open-circuit voltage signal $V_{\mathrm{PEH},\mathrm{OC}}$, respectively. If there are points in time, where the open-circuit voltage meets the perfect condition for high ${\bar{V}}_{C1}$, this will naturally result in high transmission rates. However, these transmission rates are outliers and choosing the voltage of ${\bar{V}}_{C1}$ too high will therefore result in maximum performance and minimum versatility. Hence, the system will only be functional for usecases that produce the corresponding amount of open-circuit voltages for high ${\bar{V}}_{C1}$. Since this is not the intended purpose, ${\bar{V}}_{C1}$ is chosen by the highest median value, which is 1.65Transmission/s for ${\bar{V}}_{C1}=35.7\;V$ and corresponds to a mean usable electrical power of 1.65Transmission/s·380μJ=627μW. Comparing the median values, the two-stage approach performs 1.94 times better than the one-stage approach.

## 6. Conclusions and Discussion

In this contribution, we introduced an energy autarchic asset tracking system based on the piezoelectric effect, which is solely powered by the mechanical vibrations of its own motion and functions without any batteries. Since PEHs are electromechanical systems, both the mechanical and the electrical side of the PEH have to be adapted to the desired usecase in order to be able to harvest the maximum amount of electrical power or energy.

Regarding the mechanical side of the PEH, several power-spectra of acceleration measurements were calculated. The PEHs resonance frequency ($f_{r}=33\;\mathrm{Hz}$) was designed to be able to gather ambient mechanical power effectively. Further investigations showed that PEHs produce much higher alternating open-circuit voltages compared to the desired distinct output voltages required to run low power electronic devices. To understand the meaning of this for the output power of the PEH, we performed a characterization measurement to evaluate the system behavior of the PEH varying both excitation frequency *f* and load-resistance $R_{L}$ at defined and reproduceable excitation conditions, provided by a vibration test system. Those measurement results reveal, that there is a maximum power point at the resonance frequency $f_{r}$ and the optimum ratio of ${\bar{V}}_{C1}/{\hat{V}}_{\mathrm{PEH},\mathrm{OC}}=0.5$. For excitation szenarios, that produce high open-circuit voltages, the one-stage approach provides a bad impedance matching, since the ratio of ${\bar{V}}_{C1}/{\hat{V}}_{\mathrm{PEH},\mathrm{OC}}$ accounts for only a few percent. Hence, the two-stage approach has a great possible merit by providing higher values of ${\bar{V}}_{C1}$ and therefore better electrical adaption of energy-extraction network to PEH.

The investigation of the performance of energy extraction networks and PEHs is a tedious task. Since two or more identical PEHs would influence each other and the exciting acceleration spectrum, they should not be measured simultaniously. For this reason, the performance of several former approaches, like the LV-DC/DC and the parallel SSHI extraction networks were compared to our proposed two-stage energy extraction circuit for sinusoidal and noise excitation, with respect to well defined excitation conditions provided by a vibration test system. The results demonstrated that the one-stage approach performs about two times better than the SSHI-approach for noise excitation with ${\hat{V}}_{\mathrm{PEH},\mathrm{OC}}=40\;V$. The main reason for this is that the SSHI-control produces unintended switching events, due to the noisy $V_{\mathrm{PEH}}$-signal. For the same test conditions, the two-stage approach performed about 20% better than the one-stage approach. However, it should be mentioned that the two-stage approach does not perform better than the one-stage approach for excitation conditions, which produce lower open-circuit voltages. For the proposed usecase, this is not an issue because the PEH provides higher open-circuit voltages.

In order to evaluate the performance under realistic conditions, the complete system was also tested in situ for the case of driving with the pushcart on asphalt street. In particular, the one-stage approach and the two-stage approach were tested for different values of ${\bar{V}}_{C1}$. As the results of the maximum transmission rates show, high regulated voltage $V_{C1}$ result in a very high energy extraction rate for the corresponding high excitation values. Due to the high open-circuit voltages ${\hat{V}}_{\mathrm{PEH},\mathrm{OC}}\approx 90\;V$ generated by this test scenario, the two-stage approach managed to achieve maximum transmission rates of up to 10.2Transmissions/s for the highest voltage of ${\bar{V}}_{C1}=56.2\;V$. This originates from the statistical behavior of the acceleration and the corresponding open-circuit voltage signal $V_{\mathrm{PEH},\mathrm{OC}}$. Therefore, applying the same setting of $V_{C1}$ to excitation cases with lower open-circuit voltages will result in a lower energy extraction rate. In the worst case, if $V_{C1}/V_{\mathrm{PEH},\mathrm{OC}}\geq 1$, there would be no energy extracted at all. This effect can be seen by the minimum transmission rate aquired for ${\bar{V}}_{C1}=56.2\;V$, which is the lowest of all test runs (0.22Transmissions/s).

The choice of the regulated voltage ${\bar{V}}_{C1}$ is a tradeoff between versatality and effectivity of the system and has to be chosen with respect to the expected open-circuit voltage ${\hat{V}}_{\mathrm{PEH},\mathrm{OC}}$. In this contribution, the regulated value of ${\bar{V}}_{C1}$ was defined a priori by the voltage devider $R_{1}/\left(R_{1}+R_{2}\right)$ to 35.7V, with respect to the best median transmission rate of 1.65Transmissions/s. So the two-stage approach delivers a 1.94 times higher performance than the one-stage approach, resulting in a mean usable output power of 627μW.

In future work, we want to investigate a dynamic adaption of ${\bar{V}}_{C1}$ according to the measured open-circuit voltages, by adding a sense electrode to the PEH, which delivers a reference value proportional to the open-circuit voltage of the PEH. In doing so, the regulated value of $V_{C1}$ will automatically adapt to the current usecase. Furthermore, we want to investigate the performance for other piezoelectric materials, like PVDF, which feature higher open-circuit voltages and might therefore benefit even more from the two-stage approach.

## Figures and Tables

**Table 1:** Explanation of the different electric energy extraction networks according to Figure 5 and summary of results from Figure 6 and Figure 7.

Approach Name	SSHI + Control	HV-DC/DC + Control	LV-DC/DC + Control	Max. Transm./s ${\hat{V}}_{\mathrm{PEH},\mathrm{OC}}=$ 60V;40V;25V Sinusoidal	Max. Transm./s ${\hat{V}}_{\mathrm{PEH},\mathrm{OC}}=40\;V$ Noise with VTS	Median Transm./s ${\hat{V}}_{\mathrm{PEH},\mathrm{OC}}\approx 90\;V$ In Situ: Asphalt
SSHI	enabled	disabled	enabled	4; 2; 1	0.38	-
One-stage	disabled	disabled	enabled	4; 2; 1	0.82	0.85
Two-stage	disabled	enabled	enabled	6; 2.6; 0.8	1	1.65

**Table 2:** Losses of the HV-DC/DC depicted in Figure 5 for ${\bar{V}}_{C1}=30\;V$, $V_{L2}=6.2\;V$, $V_{D2}=0.8\;V$, MOSFET BSS123 with $R_{\mathrm{DSon}}=6\;\Omega$.

Core	RM5	ER11 with Airgap	ER11 without Airgap
Comparator	LTC1540	TS881	TS881
Volume	$1360\;mm^{3}$	$318\;mm^{3}$	$318\;mm^{3}$
$t_{\mathrm{on}}$	150μs	13.3μs	13.3μs
$t_{\mathrm{Miller}}$	400ns	83ns	83ns
$L_{1}$	315mH	5.35mH	46.1mH
$L_{2}$	75mH	1.28mH	11.6mH
$R_{L1}$	5.1Ω	10.0Ω	9.9Ω
$R_{L2}$	3.15Ω	3.77Ω	3.77Ω
**Results**			
$E_{\mathrm{in}}/\mathrm{Period}$	32.1μJ	14.9μJ	1.7μJ
$E_{\mathrm{Rdson}}$	133nJ	133nJ	1.8nJ
$E_{L1}$	46.9nJ	220nJ	3.0nJ
$E_{L2}$	225nJ	670nJ	8.7nJ
$E_{D2}$	3297nJ	1526nJ	177nJ
$E_{\mathrm{swOff}}$	51nJ	56nJ	6.5nJ
**Efficiency**	89%	81%	89%

**Table 3:** Statistic results of the in situ measurement on asphalt displayed in Figure 7.

	One-Stage	Two-Stage								
${\bar{V}}_{C1}/V$	6.7	6.8	8.8	14.1	22.2	29.5	35.7	41.3	48.2	56.2
Transmissions/s										
Maximum	1.44	1.24	1.63	2.49	3.94	5.27	7.48	6.17	7.05	10.2
75th Percentile	1.00	0.87	0.97	1.37	2.05	2.31	2.22	2.64	2.37	1.91
Median	0.85	0.73	0.81	1.17	1.59	1.51	1.65	1.51	1.23	1.07
25th Percentile	0.70	0.65	0.70	1.03	1.21	1.17	1.26	1.29	0.84	0.65
Minimum	0.55	0.51	0.53	0.67	0.74	0.29	0.57	0.36	0.30	0.22

## Abbreviations

The following abbreviations are used in this manuscript:

PEH	Piezoelectric Energy Harvester
RFID	Radio Frequency Identification
VTS	Vibration Test System
MPP	Maximum Power Point
μC	Micro Controller
TX	Radio Transmitter
OOK	On Off Keying
BPSK	Binary Phase Shift Keying
acc	Acceleration
RMS	Root Mean Square value
SSHI	Synchronized Switching Harvesting with Inductor
SECE	Synchronized Electric Charge Exctraction
OC	Open-Circuit
Diff	Differentiator
LV-DC/DC	Low Voltage—Distinct Current/Distinct Current Converter
HV-DC/DC	High Voltage—Distinct Current/Distinct Current Converter

## References

[1] Swain B. (2017). Recovery and recycling of lithium: A review. Sep. Purif. Technol..

[2] Erturk A., Inman D.J. (2011). Piezoelectric Energy Harvesting.

[3] Spies P., Pollak M., Mateu L. (2015). Handbook of Energy Harvesting Power Supplies and Applications.

[4] Dorsch P., Bartsch T., Hubert F., Milosiu H., Rupitsch S.J. (2018). A Two-stage Energy Extraction Circuit for Energy Harvesting in Non-Sinusoidal Excited Environments. Proceedings.

[5] Hande A., Bridgelall R., Zoghi B. (2010). Vibration Energy Harvesting for Disaster Asset Monitoring Using Active RFID Tags. Proc. IEEE.

[6] Rupitsch S.J. (2019). Piezoelectric Sensors and Actuators—Fundamentals and Applications.

[7] Iqbal M., Khan F.U. (2018). Hybrid vibration and wind energy harvesting using combined piezoelectric and electromagnetic conversion for bridge health monitoring applications. Energy Conver. Manag..

[8] Cleante V., Brennan M., Gatti G., Thompson D. (2019). On the target frequency for harvesting energy from track vibrations due to passing trains. Mech. Syst. Signal Process..

[9] Le M.Q., Capsal J.F., Lallart M., Hebrard Y., Ham A.V.D., Reffe N., Geynet L., Cottinet P.J. (2015). Review on energy harvesting for structural health monitoring in aeronautical applications. Prog. Aerosp. Sci..

[10] Koriath H.J., Drossel W.G., Schubert A., Putz M., Wittstock V., Peter S., Hensel S., Pierer A., Müller B., Schmidt M. Experimental and numerical study on inherent sensory characteristics of piezoceramic micro parts during joining by forming in metal sheets. Proceedings of the 8th ECCOMAS Thematic Conference on Smart Structures and Materials.

[11] Kim S., Towfeeq I., Dong Y., Gorman S., Rao A., Koley G. (2018). P(VDF-TrFE) Film on PDMS Substrate for Energy Harvesting Applications. Appl. Sci..

[12] Orrego S., Shoele K., Ruas A., Doran K., Caggiano B., Mittal R., Kang S.H. (2017). Harvesting ambient wind energy with an inverted piezoelectric flag. Appl. Energy.

[13] Dorsch P., Gedeon D., Weiss M., Rupitsch S.J. Simulation-based design and optimization of piezoelectric energy harvesting systems: from mechanical excitation to usable electrical energy. Proceedings of the 2016 Joint IEEE International Symposium on the Applications of Ferroelectrics, European Conference on Application of Polar Dielectrics, and Piezoelectric Force Microscopy Workshop (ISAF/ECAPD/PFM).

[14] Dorsch P., Gedeon D., Weiß M., Rupitsch S.J. (2017). Entwicklung und Optimierung eines piezoelektrischen Energy-Harvesting-Systems zur Energieversorgung eines Güterverfolgungssystems im Logistikbereich. Tech. Messen.

[15] Robert J., Lindner T., Milosiu H. Sub 10 *μ*W wake-up-receiver based indoor/outdoor asset tracking system. Proceedings of the IEEE 20th Conference on Emerging Technologies & Factory Automation (ETFA).

[16] Tolentino I.M., Talampas M.R. Design, development, and evaluation of a self-powered GPS tracking system for vehicle security. Proceedings of the 2012 IEEE Sensors.

[17] Finkenzeller K. (2015). RFID-Handbuch: Grundlagen und Praktische Anwendungen von Transpondern, Kontaktlosen Chipkarten und NFC.

[18] Lindner T., Hafenecker S., Hadaschik N., Thielecke J. A practical evaluation of joint angle and delay estimation. Proceedings of the 2015 International Conference on Indoor Positioning and Indoor Navigation (IPIN).

[19] Gedeon D., Rupitsch S.J. (2017). Finite Element Based System Simulation for Piezoelectric Vibration Energy Harvesting Devices. J. Intell. Mater. Syst. Struct..

[20] Chew Z.J., Zhu M. (2018). Adaptive Maximum Power Point Finding Using Direct VOC/2 Tracking Method with Microwatt Power Consumption for Energy Harvesting. IEEE Trans. Power Electron..

[21] Lefeuvre E., Badel A., Richard C., Petit L., Guyomar D. (2006). A comparison between several vibration-powered piezoelectric generators for standalone systems. Sens. Actuators A Phys..

[22] Guyomar D., Lallart M. (2011). Recent Progress in Piezoelectric Conversion and Energy Harvesting Using Nonlinear Electronic Interfaces and Issues in Small Scale Implementation. Micromachines.

[23] Mateu L., Luhmann L., Zessin H., Spies P. Modified parallel SSHI AC-DC converter for piezoelectric energy harvesting power supplies. Proceedings of the 2011 IEEE 33rd International Telecommunications Energy Conference (INTELEC).

[24] Aguayo A.E., Paul O., Galchev T. Integrated synchronous electric charge extraction system for piezoelectric energy harvesters. Proceedings of the 2015 IEEE International Symposium on Circuits and Systems (ISCAS).

[25] Fan S., Wei R., Zhao L., Yang X., Geng L., Feng P.X.L. (2018). An Ultralow Quiescent Current Power Management System With Maximum Power Point Tracking (MPPT) for Batteryless Wireless Sensor Applications. IEEE Trans. Power Electron..

[26] (2010). Linear Technology Corporation.LTC 3388-1/LTC 3388-3 20 V High Efficiency Nanopower Step-Down Regulator Datasheet. https://www.analog.com/media/en/technical-documentation/data-sheets/338813fa.pdf.

[27] Roundy S., Wright P.K., Rabaey J. (2003). A study of low level vibrations as a power source for wireless sensor nodes. Comp. Commun..

[28] Stordeur M., Stark I. Low power thermoelectric generator-self-sufficient energy supply for micro systems. Proceedings of the XVI ICT ’97. Proceedings ICT’97. 16th International Conference on Thermoelectrics.

[29] Mehra A., Zhang X., Ayon A., Waitz I., Schmidt M., Spadaccini C. (2000). A six-wafer combustion system for a silicon micro gas turbine engine. J. Microelectromech. Syst..

[30] Albach T., Sutor A., Lerch R. (2009). Elektromechanischer Energiewandler auf Basis eines piezokeramischen Biegebalkens. Tech. Messen.

[31] Pi Z., Zhang J., Wen C., Zhang Z., Wu D. (2014). Flexible piezoelectric nanogenerator made of poly(vinylidenefluoride-co-trifluoroethylene) (PVDF-TrFE) thin film. Nano Energy.

[32] Kim I.H., Baik D.H., Jeong Y.G. (2012). Structures, electrical, and dielectric properties of PVDF-based nanocomposite films reinforced with neat multi-walled carbon nanotube. Macromol. Res..

[33] Xu R., Kim S.G. Figures of Merits of Piezoelectric Materials in Energy Harvesters. Proceedings of the PowerMEMS 2012.

[34] Rupitsch S.J., Lerch R. (2009). Inverse Method to estimate material parameters for piezoceramic disc actuators. Appl. Phys. A.

[35] Rupitsch S.J., Wolf F., Sutor A., Lerch R. (2012). Reliable modeling of piezoceramic materials utilized in sensors and actuators. Acta Mech..

[36] Rupitsch S.J., Ilg J. (2015). Complete characterization of piezoceramic materials by means of two block-shaped test samples. IEEE Trans. Ultrason. Ferroelectr. Freq. Control.

[37] Weiß M., Ilg J., Rupitsch S.J., Lerch R. (2016). Inverse Methode zur Charakterisierung des mechanischen Frequenzverhaltens isotroper Werkstoffe. Tech. Messen.

[38] Ramadass Y.K. (2009). Energy Processing Circuits for Low-Power Applications. Ph.D. Thesis.

[39] Mateu Sáez M.L., Luehmann L., Babel P., Pollak M., Spies P. (2013). Peak Detector For Switched AC/DC Converter.

[40] Mateu Sáez M.L., Luehmann L., Babel P., Pollak M., Spies P. (2016). Rectifier Circuit with AC Side Short-Circuiting Functions and Synchronized Switch Harvesting on Inductor Converter. U.S. Patent.

[41] Erturk A., Inman D.J. (2008). Issues in mathematical modeling of piezoelectric Energy Harvesters. Smart Mater. Struct..

[42] Thorsten Hehn Y.M. (2014). CMOS Circuits for Piezoelectric Energy Harvesters.

[43] Shu Y.C., Lien I.C. (2006). Efficiency of energy conversion for a piezoelectric power harvesting system. J. Micromech. Microeng..

